# Implementation of a billable transitional care model for stroke patients: the COMPASS study

**DOI:** 10.1186/s12913-019-4771-0

**Published:** 2019-12-19

**Authors:** Sabina B. Gesell, Cheryl D. Bushnell, Sara B. Jones, Sylvia W. Coleman, Samantha M. Levy, James G. Xenakis, Barbara J. Lutz, Janet Prvu Bettger, Janet Freburger, Jacqueline R. Halladay, Anna M. Johnson, Anna M. Kucharska-Newton, Laurie H. Mettam, Amy M. Pastva, Matthew A. Psioda, Meghan D. Radman, Wayne D. Rosamond, Mysha E. Sissine, Joanne Halls, Pamela W. Duncan

**Affiliations:** 1 Department of Social Sciences and Health Policy, Department of Implementation Science, Wake Forest School of Medicine, One Medical Center Boulevard, Winston-Salem, NC 27157 USA; 2 Department of Neurology, Wake Forest School of Medicine, Winston-Salem, NC USA; 3 Department of Epidemiology, University of North Carolina at Chapel Hill, Gillings School of Global Public Health, Chapel Hill, NC USA; 4 Department of Biostatistics, University of North Carolina at Chapel Hill, Gillings School of Global Public Health, Chapel Hill, NC USA; 5 Department of Family Medicine, University of North Carolina at Chapel Hill, Chapel Hill, NC USA; 6 University of North Carolina at Wilmington, School of Nursing, Wilmington, NC USA; 7 Duke University, School of Medicine, Durham, NC USA; 8 University of Pittsburgh, School of Health and Rehabilitation Sciences, Pittsburgh, PA USA; 9 Department of Epidemiology, University of Kentucky, College of Public Health, Lexington, KY USA; 10 Department of Earth and Ocean Sciences, University of North Carolina at Wilmington, Wilmington, NC USA

**Keywords:** Implementation Science, Stroke, Ischemic Attack, Transient, Transitional Care, Reimbursement Mechanisms

## Abstract

**Background:**

The COMprehensive Post-Acute Stroke Services (COMPASS) pragmatic trial compared the effectiveness of comprehensive transitional care (COMPASS-TC) versus usual care among stroke and transient ischemic attack (TIA) patients discharged home from North Carolina hospitals. We evaluated implementation of COMPASS-TC in 20 hospitals randomized to the intervention using the RE-AIM framework.

**Methods:**

We evaluated hospital-level Adoption of COMPASS-TC; patient Reach (meeting transitional care management requirements of timely telephone and face-to-face follow-up); Implementation using hospital quality measures (concurrent enrollment, two-day telephone follow-up, 14-day clinic visit scheduling); and hospital-level sustainability (Maintenance). Effectiveness compared 90-day physical function (Stroke Impact Scale-16), between patients receiving COMPASS-TC versus not. Associations between hospital and patient characteristics with Implementation and Reach measures were estimated with mixed logistic regression models.

**Results:**

Adoption: Of 95 eligible hospitals, 41 (43%) participated in the trial. Of the 20 hospitals randomized to the intervention, 19 (95%) initiated COMPASS-TC.

Reach: A total of 24% (656/2751) of patients enrolled received a billable TC intervention, ranging from 6 to 66% across hospitals.

Implementation: Of eligible patients enrolled, 75.9% received two-day calls (or two attempts) and 77.5% were scheduled/offered clinic visits. Most completed visits (78% of 975) occurred within **14 days**.

Effectiveness: Physical function was better among patients who attended a 14-day visit versus those who did not (adjusted mean difference: 3.84, 95% CI 1.42–6.27, *p* = 0.002).

Maintenance: Of the 19 adopting hospitals, 14 (74%) sustained COMPASS-TC.

**Conclusions:**

COMPASS-TC implementation varied widely. The greatest challenge was reaching patients because of system difficulties maintaining consistent delivery of follow-up visits and patient preferences to pursue alternate post-acute care. Receiving COMPASS-TC was associated with better functional status.

**Trial registration:**

ClinicalTrials.gov number: NCT02588664. Registered 28 October 2015.

## Background

Stroke is the fifth leading cause of death in the United States (US) and the leading preventable cause of disability [1]. Significant improvements in acute stroke care over the past two decades have reduced mortality and increased the number of patients that need assistance transitioning to their communities in the presence of complex comorbidity and residual deficits. Substantial evidence supports the use of stroke rehabilitation and secondary prevention after stroke [2], and growing evidence supports transitional care (TC) models that have since become the standard of care in other countries [3, 4]. However, in the US, there is still no standard of care for stroke patients discharged home [5, 6]. Instead, post-acute care for stroke patients discharged home is fragmented and poorly coordinated and lacks continuity. To encourage providers to offer transitional care management (TCM) services to patients discharged home, the Centers for Medicare and Medicaid Services (CMS) created billing codes for reimbursement. However, TCM billing remains underutilized (7% in 2015) [7].

The large, pragmatic, cluster-randomized COMprehensive Post-Acute Stroke Services (COMPASS) Study examined the comparative effectiveness of a comprehensive TC model (COMPASS-TC) versus usual care for individuals discharged home following a stroke or transient ischemic attack (TIA). COMPASS-TC included evidence-based components of early supported discharge (ESD) [4, 8] and met CMS TCM reimbursement requirements. The primary results of the COMPASS trial have been described [9]. Importantly, we observed considerable heterogeneity in delivery of COMPASS-TC, which suggests that there are hospital-level factors that drive implementation and warrant further investigation.

Given the importance of healthcare redesign to provide more effective and sustainable chronic disease management, it is critical to understand the factors that describe which hospitals were most likely to deliver an intervention that meets TCM billing requirements and which patients were most likely to receive it. This will inform future efforts to implement TC and use TCM billing codes. The aim of this implementation study was to leverage the experiences from the COMPASS Study to evaluate the first phase of implementation of a billable TCM model for patients discharged home with stroke or TIA in the context of real-world clinical practice across the state of North Carolina (NC).

## Methods

### Study design and sites

The COMPASS study design and selection of hospitals are described in detail elsewhere [10, 11]. Briefly, hospitals were randomized to either the intervention (COMPASS-TC) or control (usual care) arm in Phase 1. In Phase 2, usual care hospitals crossed over to implement the intervention, and the original intervention hospitals attempted to sustain COMPASS-TC with limited study support.

In this paper we analyzed implementation of COMPASS-TC in the 20 hospitals randomized to the intervention arm in Phase 1, including 2751 patients enrolled July 2016 through March 2018 [12]. We evaluated patient and hospital characteristics associated with successful implementation. Our reporting follows the Standards for Reporting Implementation Studies (StaRI) guidelines for transparent and accurate reporting of implementation studies [13] and adheres to CONSORT guidelines.

### Context: hospital engagement

As required by the Patient-Centered Outcomes Research Institute (PCORI), hospitals used their existing infrastructure, budget, and staffing to deliver the intervention. The study paid hospitals $50 per enrolled patient (plus $105 per patient who returned to the COMPASS follow-up clinic and received a care plan within 18 days of discharge) but did not pay for staff time to deliver the intervention. Hospitals also received $14,000 prior to implementation to offset costs of training and building infrastructure. These payments were not meant to cover costs. In line with pragmatic trial design as well as PCORI funding guidelines, the financial assistance to hospitals was minimal [14]. The study did not provide financial assistance for intervention costs including compensation for personnel that were delivering the intervention and equipment and materials costs associated with delivery of the intervention. COMPASS-TC was integrated into patient care without additional personnel resources provided to hospitals [10].

### Study sample

This implementation analysis included all data from patients enrolled in the Phase 1 intervention arm (*n* = 2751 events among *n* = 2689 patients). Patients were eligible for enrollment if they were aged 18 years or older, spoke English or Spanish, were diagnosed with ischemic stroke, hemorrhagic stroke (excluding subdural or aneurysmal hemorrhage), or TIA, and were discharged directly home [10].

### COMPASS-TC as a billable TCM intervention

The COMPASS-TC intervention was designed to be consistent with CMS TCM reimbursement requirements [15–17], which include requiring that the patient:

- Transition from an inpatient setting (e.g., acute care hospital) to home;
- Be of moderate- or high-complexity medical decision-making;
- Receive communication (direct contact, telephone, or electronic) within two business days of discharge or two or more documented attempts within two business days; and
- Have a face-to-face clinical visit within seven calendar days (for CPT Code 99496) or within 14 calendar days (for CPT Code 994965) of discharge from the inpatient setting [17].

The foundational component of the COMPASS-TC intervention was post-acute care coordination and management by a registered nurse (RN), post-acute care coordinator (PAC) and an advanced practice provider (APP), defined as a nurse practitioner (NP), physician assistant (PA), or physician. The PAC and/or APP contacted the patient in-person before hospital discharge to home, by phone two days post-discharge (or two attempts), and saw the patient in-person at the COMPASS-TC clinic visit within 14 days of discharge. During the pre-discharge visit, the PAC/APP described COMPASS-TC to the patient and family. The hospital-based team utilized an electronic TC planning tool developed by the study team to systematically evaluate patients [15] during both telephone and face-to-face follow-up. During the two-day call, the PAC/APP asked if any cognitive or physical deficits became apparent at home, completed medication review and reconciliation, referred to home health or outpatient care if indicated, provided patient education stroke symptoms, and reminded the patient of the upcoming clinic visit. Hospitals had flexibility in their COMPASS clinic setting (neurology clinic, hospital-based non-specialty clinic, primary care provider (PCP) clinic). During the clinic visit, the PAC/APP performed a standardized assessment of the patient’s functional status, medical and neurological care needs, social determinants of health, and caregiver’s capacity for assisting the patient during recovery [15, 16]. The results of the comprehensive assessment were captured electronically and generated an individualized electronic care plan (eCare Plan) that was shared with the patient, caregiver, PCP, and home health and outpatient therapy where applicable. The eCare Plan identified areas of patient/caregiver need and directed the PAC/APP in appropriate referrals to relevant community-based services (e.g., caregiver support services, medication management). Each hospital, in partnership with the study team, assembled a community resource directory of available services in their county to populate the eCare Plan, and a network of service providers in their area (community resource network) to support these referrals.

### Implementation strategies

An implementation strategy is defined as an activity that facilitates adoption, implementation, and sustainability [18]. We utilized a number of implementation strategies [19] prior to hospital implementation and during active implementation of COMPASS-TC (Additional file 2: Table S1). After hospitals were randomized, training for intervention hospitals consisted of: two-day intensive training “boot camp” (to explain the care model), 6-h site visit (to tailor implementation, identify available community resources, and build community resource networks (CRN)), bi-monthly peer problem-solving calls, monthly data feedback on performance, and one-on-one same-day consulting as requested. Training and ongoing consultation was provided to all intervention hospitals by the study’s Director of Implementation who had both clinical and administrative experience, and a team of multidisciplinary providers with experience in stroke care. All training materials were approved by stakeholders, including patients and caregivers. Educational and training modules were made available online, and monthly educational webinars were provided on a variety of stroke/TIA topics relevant to clinical practice.

### RE-AIM framework & measures

To assess implementation, we used the RE-AIM framework, the most widely used framework in implementation science for evaluation [20]. RE-AIM evaluates the Reach, Effectiveness, Adoption, Implementation, and Maintenance of health promoting interventions in real-world, complex settings, attending to both individual and organizational levels of impact, to address questions of translation into practice and generalizability. This framework focuses on features of the settings and participants, features of the implementers, and the frequency and intensity of intervention activities [13] and thus is suitable for process evaluations.

***Reach*** assesses who received the intervention [21] and is a patient-level measure of participation [12]. Randomization occurred at the hospital level. The study met criteria for a waiver of consent and HIPAA Authorization; therefore all patients were enrolled automatically and given opportunities to decline participation in the outcomes survey or to withdraw from the research study entirely [22]. Hospital staff screened, identified, and enrolled eligible patients and initiated the intervention. All patients in the study were theoretically eligible for TCM at discharge as they all transitioned from the hospital directly home with a diagnosis of stroke or TIA, which meet the criteria for moderate- or high-complexity medical decision making. Thus, successfully “reached” patients were those whose care met all TCM billing requirements, including receipt of a call two business days post-discharge (or two attempts) and attendance at a clinic visit (which includes an eCare Plan) 14 calendar days post-discharge. Reach was calculated as the proportion of reached patients out of the total enrolled by hospital staff.

***Effectiveness*** assesses what effect the intervention had on important outcomes [21]. The primary outcome for the COMPASS Study was physical function measured with the Stroke Impact Scale-16 (SIS-16) [23]. The Effectiveness metric was defined as within-hospital mean differences in SIS-16 between patients receiving a 14-day clinic visit versus not. A comparison of COMPASS-TC versus usual care has been presented elsewhere [9].

***Adoption*** assesses where the intervention was implemented [21]. The Adoption metrics were: the number and proportion of hospitals that initiated the intervention, number and characteristics of intervention agents (clinical team delivering the intervention: PAC, PAC back-up, APP, and APP back-up) that were trained, time it took to launch after training, and number of intervention agents that delivered the intervention.

***Implementation*** assesses how each component of the intervention was delivered [21] as intended, including time needed for the implementation [12] of the clinic visit, which is the core of the COMPASS-TC model. The Implementation metrics were defined as:

1. Fidelity to each of the components of the intervention measured with quality measures that were created as part of the COMPASS Study to provide real-time feedback to hospitals (Appendix for details):

- Proportion of patients identified and enrolled within 2 days,
- Proportion of patients who had a two-day call or documentation of two attempts (i.e., delivered per protocol),
- Proportion of patient scheduled/offered a clinic visit within 14 days, even if patient did not attend (i.e. delivered per protocol),
- Proportion of all completed visits occurring within 14 days, and
- Proportion of all completed visits during which an eCare Plan generated.

(2) Clinic visit duration (minutes)
(3) Number of days from discharge to clinic visit among participants who attended a visit at any time.

***Maintenance*** assesses how long the intervention was sustained. The maintenance metric was defined as the absolute number, proportion, and characteristics of hospitals that continued to enroll patients and deliver components of COMPASS-TC for a minimum of 6 months after the end of Phase 1.

### Data collection

Process data, such as dates of completion and reasons for not completing a study-related task, were used to compute ***quality measures****.* These were collected during the study by hospital staff using forms embedded in a web-based application.

***Hospital characteristics*** were obtained from public data files (e.g., Rural-Urban Community Area (RUCA) code website [24], Joint Commission website [25], NC Stroke Care Collaborative hospital characteristics, Medicare Provider of Services files) [26], and baseline hospital surveys [10, 27], which ascertained staff turnover, dates of training, and clinic locations. Additional surveys administered approximately 6 months into the study captured clinic visit duration, organizational readiness, and partnership synergy. Organizational readiness was measured by the Organizational Readiness to Implement Change (ORIC) Scale [28], which measures change commitment and change efficacy. ORIC is a validated instrument that was administered to the intervention agents (clinical team delivering the intervention: PAC, PAC back-up, APP, and APP back-up). The Partnership Synergy Scale measured the level of engagement between the clinical team and their CRN, as a proxy for how much the community network assisted PACs with linking patients to community-based social services [29]. This is a validated instrument and was administered to intervention agents, site principal investigator, hospital leadership, and community network members (e.g., engaged community-based pharmacists, social service providers, rehabilitation providers supporting the PAC in linking patients to needed services and resources outside the hospital).

***Patient demographic and clinical characteristics*** were obtained from electronic health records and recorded by hospital staff. Patient addresses were geocoded using ArcMap 10.5.1 and the World Geocoding Service. Three (0.1%) patients did not have home addresses and were not geocoded. Shortest distance to the COMPASS clinic visit location was computed using Open Streetmap and ArcGIS Network Analyst.

***Patient outcomes*** were obtained at 90 days by trained (blinded) interviewers administering a phone survey that included self-reported post-stroke physical function, measured with the Stroke Impact Scale-16 (SIS-16) [23]. The SIS-16 was selected as the primary patient outcome for the COMPASS study because of its strong psychometric properties, including validation for proxy, phone, and mail administration [30, 31]. It is a patient-centric measure, developed in with input from patients, caregivers, and providers [23]. It was designed to capture significant residual deficits in mild and moderate stroke, and is superior to other measures in capturing residual deficits that matter to patients. Even in those with the mildest strokes (NIH Stroke Severity Score 0 to 5), only 10% report full function on the SIS-16. The 16 items measure ADLS, IADLS, and physical activities on a scale of 0–100, with higher scores indicating higher function. Patient outcomes were collected by interviewers at the Carolina Survey Research Laboratory. Interviewers were trained on study-specific protocols that incorporated patient feedback obtained during pilot testing. Interviewers were blinded to randomization arm and administered all interviews using standardized computer-assisted telephone interviewing software and scripts [10]. Interviewers were monitored biweekly by their supervisors for quality control.

***Hospital audits*** Hospitals participated in two unannounced case ascertainment audits, each covering a 2 month period, to evaluate the proportion of eligible cases that hospitals correctly identified and enrolled.

***Consent*** Institutional review board (IRB) approval was received through Wake Forest University Health Sciences (central IRB), or through local hospital IRBs. At 90 days, patients provided informed consent [22] for collection of outcomes. Additionally, participants consented to collection of process measures, including the ORIC and Partnership Synergy Scale via email survey.

### Statistical analyses

Descriptive statistics were used to summarize each RE-AIM domain. Characteristics of enrolled patients who were reached versus not were compared using Fisher’s exact and Wilcoxon rank sum tests. Generalized linear mixed models (GLMMs) were used to evaluate patient characteristics associated with clinic visit attendance (a primary component of patient Reach) and were adjusted for clinic setting, distance to the clinic, and organizational readiness. Analyses were performed conditional on successful implementation of the 14-day visit (i.e., a visit being scheduled or offered to the patient).

Associations between hospital characteristics and both Reach and Implementation quality measures were estimated with GLMMs, adjusted for patient characteristics including age, race, gender, diagnosis, stroke severity, history of stroke or TIA, presence of at least one cardiovascular comorbidity (i.e., cardiovascular disease, heart failure, atrial fibrillation, or diabetes), and distance to the COMPASS-TC follow-up clinic.

Linear mixed models were used to estimate overall and hospital-specific mean differences in SIS-16 [23] between patients who received a visit and eCare plan within 14 days compared with those who did not receive a visit (Effectiveness). These models were adjusted for age, race, stroke severity, diagnosis, and a log-transformed patient-specific propensity score. Propensity scores were constructed to account for differences in patient characteristics of those who did and did not attend the clinic visit. Propensity scores were estimated with conditional logistic regression and incorporated information such as medical history and comorbidity [9].

Finally, *p*-values for associations between hospital characteristics and Maintenance were obtained using Fisher’s Exact and Wilcoxon Rank Sum tests.

***Missing Data*** Some covariate data were incompletely ascertained (e.g., 2% missing stroke severity). Multiple imputation with chained equations [32] was used to construct 100 complete datasets that were analyzed with GLMMs as described above and estimates were combined using standard techniques [33].

## Results

**Fig. 1:**
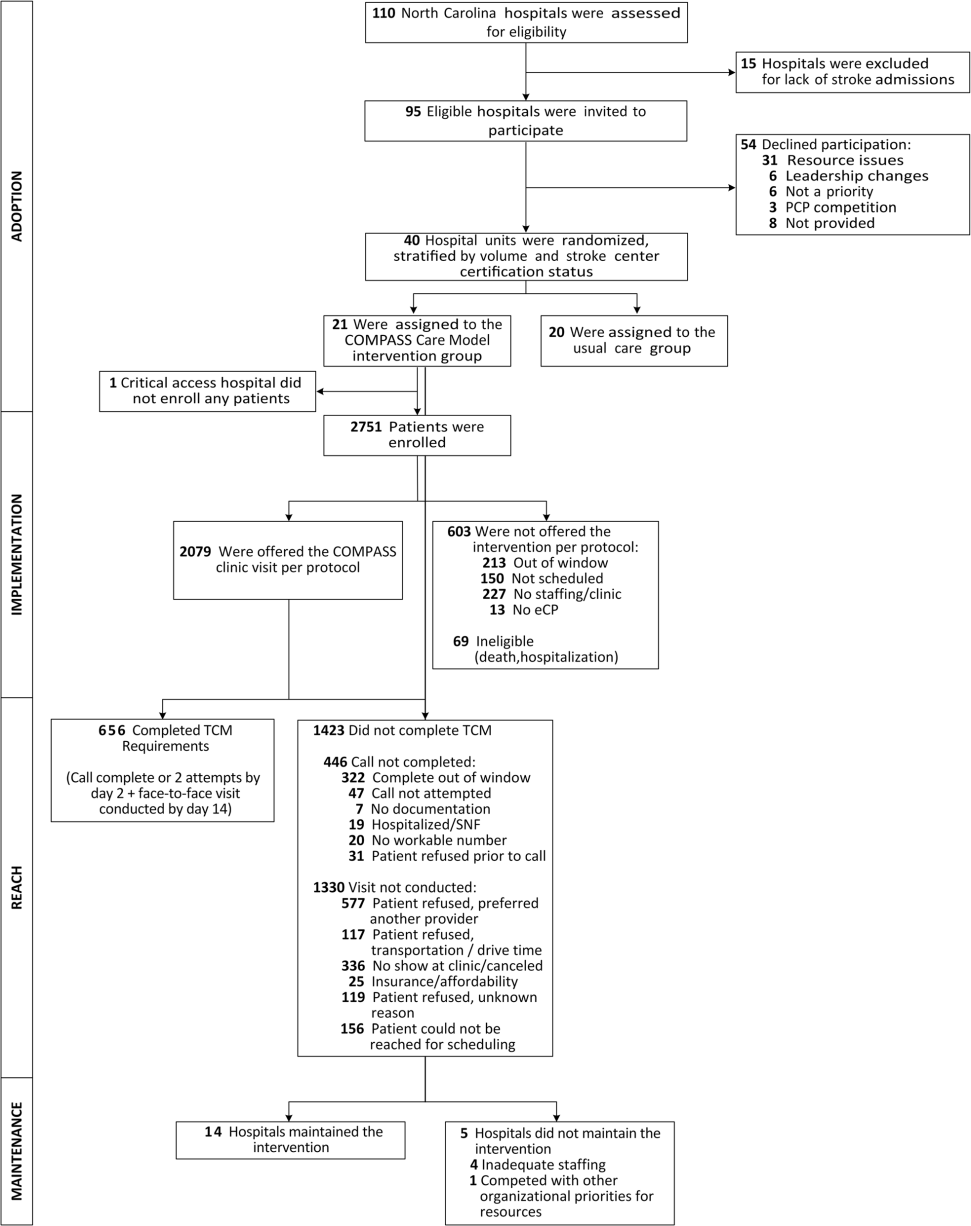
Extended CONSORT Flow Diagram

**Fig. 2:**
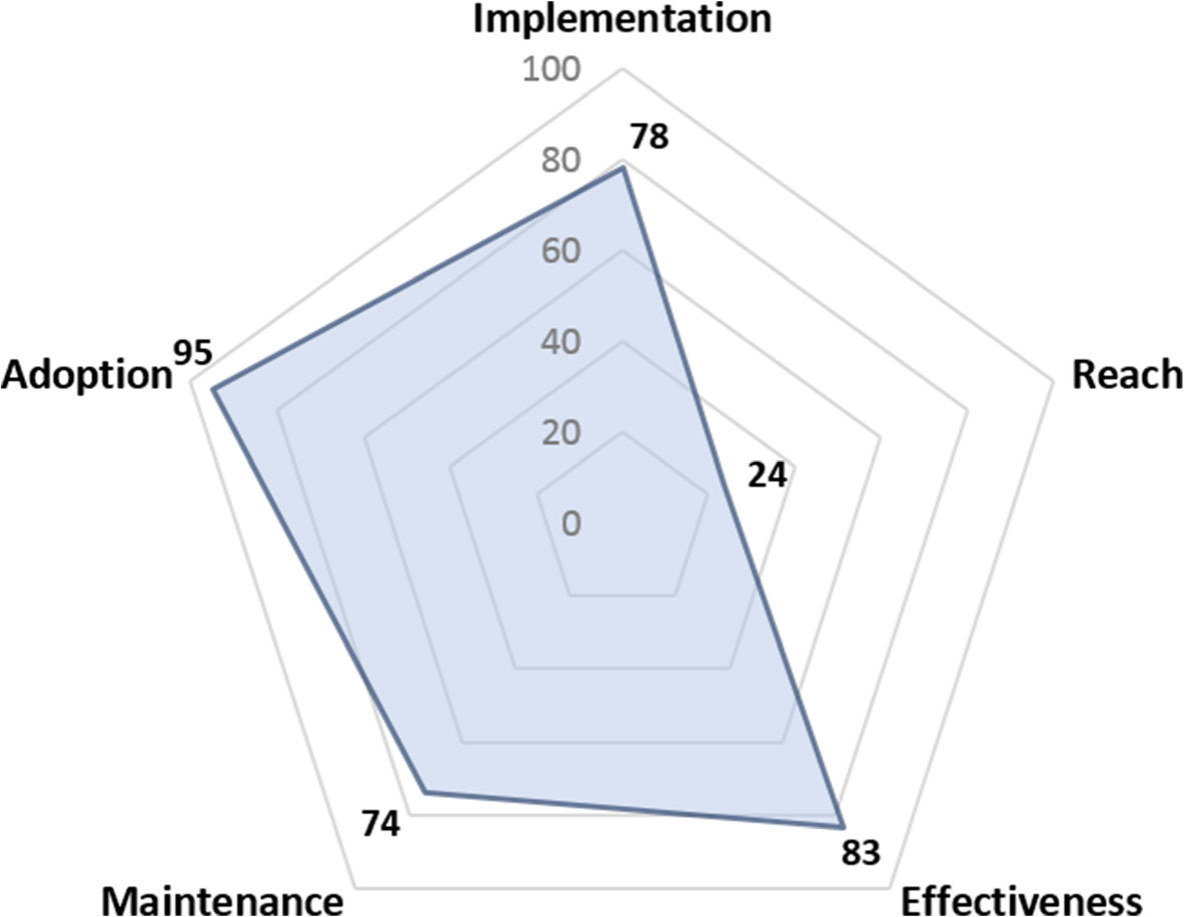
At-A-Glance Summary of Implementation of COMPASS-TC. Effectiveness is shown as the unadjusted mean Stroke Impact Scale (SIS)-16 (on a scale of 0–100) among patients that received COMPASS-TC within 14 days. These patients had an average adjusted score ~ 4-points higher than patients with no visit

The extended CONSORT diagram [34] provides summary information about each RE-AIM dimension in temporal order, beginning with adoption (Fig. 1). Taken together, hospital-level adoption of the intervention was moderate among all eligible hospitals, and high among enrolled hospitals. Implementation and Maintenance were high, and the effectiveness of treatment was largely consistent across hospitals; however, Reach was low (Fig. 2). Results for each RE-AIM dimension are described in detail below.

### Adoption

Ninety-five hospitals were eligible and invited to participate, of which 41 (43%) signed a letter of agreement to participate, received IRB approval, and were randomized in 40 units. Details of hospital recruitment have been published [11]. Participating hospitals, compared to non-participating hospitals, were more likely to be Primary Stroke Centers (59% vs. 41%), located in metropolitan areas (54% vs. 45%), and have high stroke volume (29% vs. 17% having ≥300 stroke discharges per year) [11].

Of the 20 hospitals randomized to the intervention arm, 19 initiated the intervention (95%). The non-adopting site was a rural, critical access hospital. Reasons for non-adoption included having a small number of stroke patients (reported average of 7 per year) and a change in executive leadership after the letter of agreement was signed.

All hospitals (20/20; 100%) completed both required trainings: an intensive two-day boot camp for intervention agents and a six-hour site visit for intervention agents and hospital and community stakeholders. The PAC role was filled by RNs except for one PA. The APP role was filled by NPs (*N* = 10), PAs (*N* = 7), and Neurologists (*N* = 2). Only 10 hospitals had a designated back-up PAC, and 5 had a designated back-up APP. At the on-site six-hour training, hospitals had a median of 7 hospital staff attendees (IQR 5–8).

Median time from training to launch was 60 days (range 25–190; IQR [39–74]). Mean full time equivalent positions to deliver the intervention was 2.15 (1.64 for low-volume [< 300 patients per year] hospitals and 2.83 for high-volume [300+ patients per year] hospitals).

### Implementation

Clinic setting for in-person follow-up varied according to the availability of staffing in each hospital and changed over time for 21% (4/19) of hospitals. Neurology-based clinics (both in-hospital and ambulatory settings) were the most common (12 hospitals, 69% of patients). Five hospitals utilized hospital-based non-specialty clinics, and 7 utilized PCP-based clinics. The median hospital organizational readiness score was 4.3 and ranged from 1.95 to 5.0, with 5 indicating most ready. The median partnership synergy score was 3.8 and ranged from 2.25 to 5.0, with 5 indicating that the hospital staff and their CRN partners collaborated to a large extent to respond to patients’ needs when transitioning home.

**Fig. 3:**
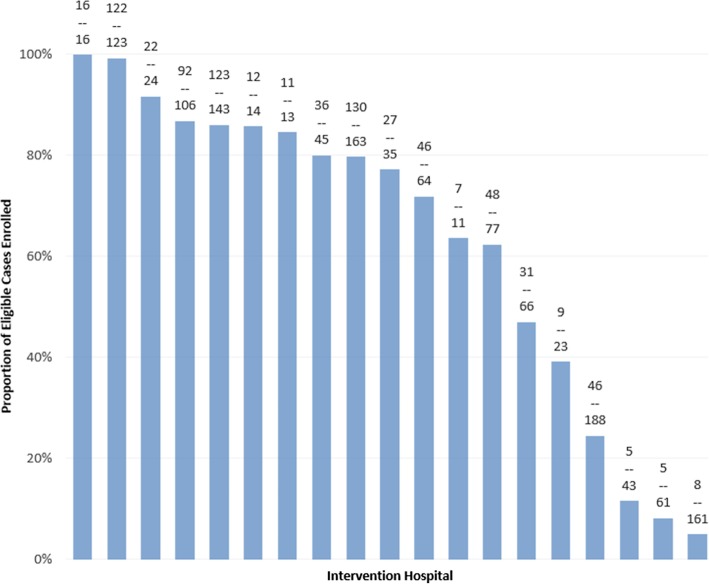
Hospital-Specific Case Ascertainment. Bars represent the proportion of eligible patients enrolled at individual hospitals over the 4 months of case ascertainment audits. The numbers of patients enrolled out of all eligible patients during the audit period are indicated above each bar

***Hospital performance on case ascertainment and enrollment*** Within the 4-month audit period, 58% (796/1376) of eligible patients were enrolled [9], with wide variability (5 to 100%) across intervention hospitals (Fig. 3). Enrolled patients were typical of those discharged directly home after a stroke [9]. The most common reason hospital staff reported for not ascertaining and enrolling cases was COMPASS staffing challenges (e.g., shortages, turnover). Other reasons included patient discharge over the weekend or while the PAC was out of the office or having been missed in screening (e.g., no presumptive stroke diagnosis, discharged directly from emergency department). Among enrolled patients, 47% were provided an introduction to the study in person before discharge, while the remainder were contacted post-discharge by phone or mail. Fifty-eight percent of patients were scheduled for a follow-up visit before discharge.

***Hospital performance on quality measures*** On average, 41.7% (*n* = 2751) of patients were screened, identified, and enrolled concurrent with care (within 2 days of discharge), and 75.9% received a two-day call (or two attempts). Further, 77.5% of eligible patients were scheduled for or offered a clinic visit. Among 975 completed clinic visits occurring at any time after discharge, 98.5% included an eCare Plan, 78% occurred within **14 days as required for CPT Code 99496**5, and 28% **within 7 days as required for CPT Code 99496** (Additional file 1: Figure S1). Shortly after implementation began, PACs estimated that, on average, they spent 43 min (SD 21 min) with patients at the clinic visit. Six months later, they reported an average 29 min (SD 17 min).

In adjusted analysis, higher organizational readiness was associated with higher odds of scheduling a clinic visit (OR = 1.60; 95% CI 1.00 to 2.58; Table 1). High volume and urban location were associated with lower odds of scheduling a clinic visit. Neurology clinics had lower odds of scheduling than hospital-based non-specialty clinics or PCP-based clinics (Table 1).

**Table 1:** Associations Between Hospital Characteristics and Implementation and Reach

	Implementation: Scheduling 14-day Clinic Visit			Reach: TCM Billing Criteria Met		
Characteristic (no. of patients)	N (%) meeting metric	OR	(95% CI)	N (%) meeting metric	OR	(95% CI)
Primary Stroke						
Center Certification						
PSC (*n* = 2270)	1713 (77.3)	0.96	(0.38, 2.41)	524 (30.6)	0.75	(0.31, 1.81)
Not PSC (*n* = 481)	366 (78.7)	1.00	(Reference)	128 (35.0)	1.00	(Reference)
Annual Stroke Volume						
300+ patients (*n* = 1643)	1173 (77.3)	0.51	(0.21, 1.23)	323 (27.5)	0.78	(0.32, 1.90)
< 300 patients (*n* = 1108)	906 (83.6)	1.00	(Reference)	329 (36.3)	1.00	(Reference)
Geographic Region						
Urban (*n* = 2405)	1755 (75.0)	0.19	(0.07, 0.53)	479 (27.3)	0.58	(0.19, 1.81)
Non-urban (*n* = 346)	324 (94.5)	1.00	(Reference)	173 (53.4)	1.00	(Reference)
Clinic Setting						
PCP (*n* = 389)	318 (84.1)	2.02	(0.82, 5.02)	71 (22.3)	0.78	(0.32, 1.89)
Hospital-based (*n* = 461)	358 (79.0)	1.70	(0.64, 4.56)	68 (19.0)	0.69	(0.26, 1.84)
Neurology (*n* = 1901)	1403 (75.8)	1.00	(Reference)	513 (36.6)	1.00	(Reference)
Organizational Readiness^ab^		1.60	(1.00, 2.58)		1.25	(0.75, 2.07)
0–3.9	759 (71.7)			166 (21.9)		
4.0–5.0	1320 (81.3)			486 (36.8)		
Partnership Synergy^ac^		1.13	(0.61, 2.08)		1.38	(0.77, 2.48)
0–3.9	951 (75.2)			203 (21.3)		
4.0–5.0	1128 (79.5)			449 (39.8)		

OR were estimated using mixed logistic regression models adjusted for age, sex, race, diagnosis, insurance status, history of stroke/TIA, presence of cardiovascular-related comorbidity, and stroke severity

Abbreviations: *OR* Odds Ratio, *PSC* Primary Stroke Center, *PCP* Primary Care Practice

^a^ OR is for 1-unit increase. Subgroups based on a cut-point of 4 are shown to summarize the number and proportion of patients meeting each metric for different levels of readiness and synergy

^b^ Organizational Readiness to Implement Change Scale was used to measure change commitment and efficacy [27]

^c^ Partnership Synergy Scale was used to measure the level of engagement between the post-acute coordinator and the community resource network [28]

### Reach

**Fig. 4:**
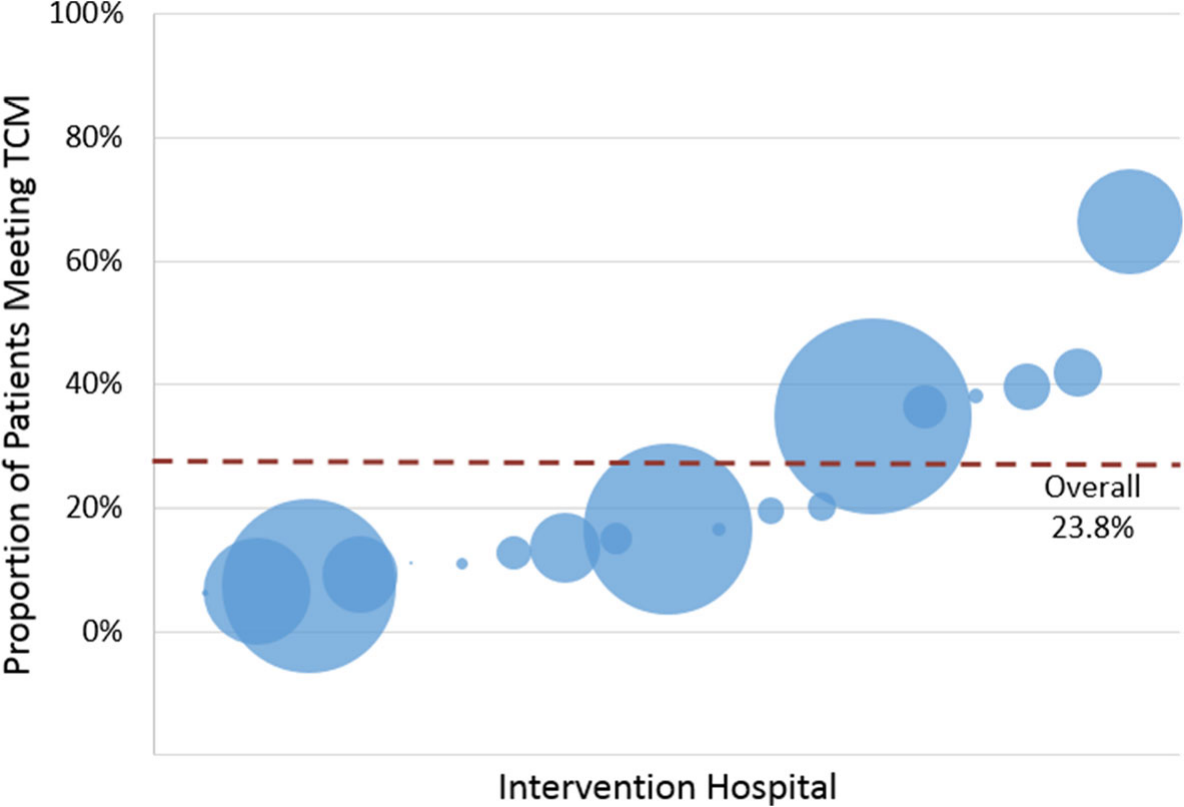
Proportion of Patients Meeting Transitional Care Management (TCM) Criteria by Hospital. Circles represent the 19 hospitals that adopted the intervention and are scaled to represent the total number of enrolled participants. Values on the y-axis represent the proportion of patients that met TCM billing criteria

A total of 656/2751 (24%) of patients enrolled at intervention hospitals received care under the COMPASS-TC model that met TCM billing code requirements. This ranged from 6 to 66% across hospitals (Fig. 4). Not receiving a 14-day clinic visit, independent of whether or not it was scheduled, was the primary reason patients were not reached (see CONSORT diagram Fig. 1 for reasons why). Of those not reached, 977 (69%) received the two-day call but did not attend the clinic visit, 353 (25%) failed to receive both the call and visit, and 93 (7%) had the visit but no call. Reach was lower at both PCP-based clinics and hospital-based non-specialty clinics compared with neurology-based clinics (Table 1). Urban hospitals had lower Reach compared with non-urban hospitals. Patients introduced to the intervention before discharge were more frequently reached than those notified post-discharge by mail or phone (34% vs. 15%, *p* < 0.0001), as were those with a clinic visit appointment scheduled prior to discharge versus not (32% vs. 12%, *p* < 0.0001).

Patient characteristics independently associated with clinic visit attendance when one was offered or scheduled included diagnosis with stroke versus TIA (OR = 1.64, 1.29 to 2.08) and mild stroke severity (Table 2). Having a history of previous stroke or TIA, lack of insurance, living farther from the clinic, and urban residence were associated with lower odds of clinic visit attendance (Table 2). Age, gender, race, and presence of a cardiovascular-related comorbidity were not strongly associated with odds of clinic visit attendance in this analysis model that also adjusted for lack of insurance, rural residence, and other characteristics.

**Table 2:** Patient Characteristics Associated with Clinic Visit Attendance within 14 Days

	OR	(95% CI)
Age (10-y increase)	0.94	0.87–1.02
Gender		
Female	0.97	0.79–1.18
Male	1.00	(Reference)
Race		
Non-white	1.09	0.81–1.46
White	1.00	(Reference)
Insurance status		
Uninsured	0.73	0.49–1.07
Insured	1.00	(Reference)
Geographic area of residence		
Urban	0.72	0.50–1.03
Non-urban	1.00	(Reference)
Diagnosis		
Stroke	1.64	1.29–2.08
TIA	1.00	(Reference)
NIH Stroke Scale Score		
0	1.00	(Reference)
1–4	1.17	0.94–1.47
5–42	0.84	0.60–1.17
History of stroke or TIA		
Yes	0.76	0.60–0.97
No	1.00	(Reference)
Presence of any cardiovascular comorbidity		
Yes	0.95	0.77–1.16
No	1.00	(Reference)
Distance to clinic		
< 24 km (< 15 miles)	1.00	(Reference)
24–47 km (15–29 miles)	0.85	0.66–1.10
48–95 km (30–59 miles)	0.71	0.50–1.00
96+ km (60+ miles)	0.33	0.20–0.54

ORs were estimated using a mixed logistic regression model that included age, sex, race, insurance status, diagnosis, history of stroke/TIA, presence of cardiovascular-related comorbidity, stroke severity, urban residence, distance to the clinic, organizational readiness, and clinic setting. Analysis included participants that were offered or scheduled a clinic visit

Abbreviations: *OR* Odds Ratio, *TIA* transient ischemic attack, *km* kilometers

### Effectiveness

**Fig. 5:**
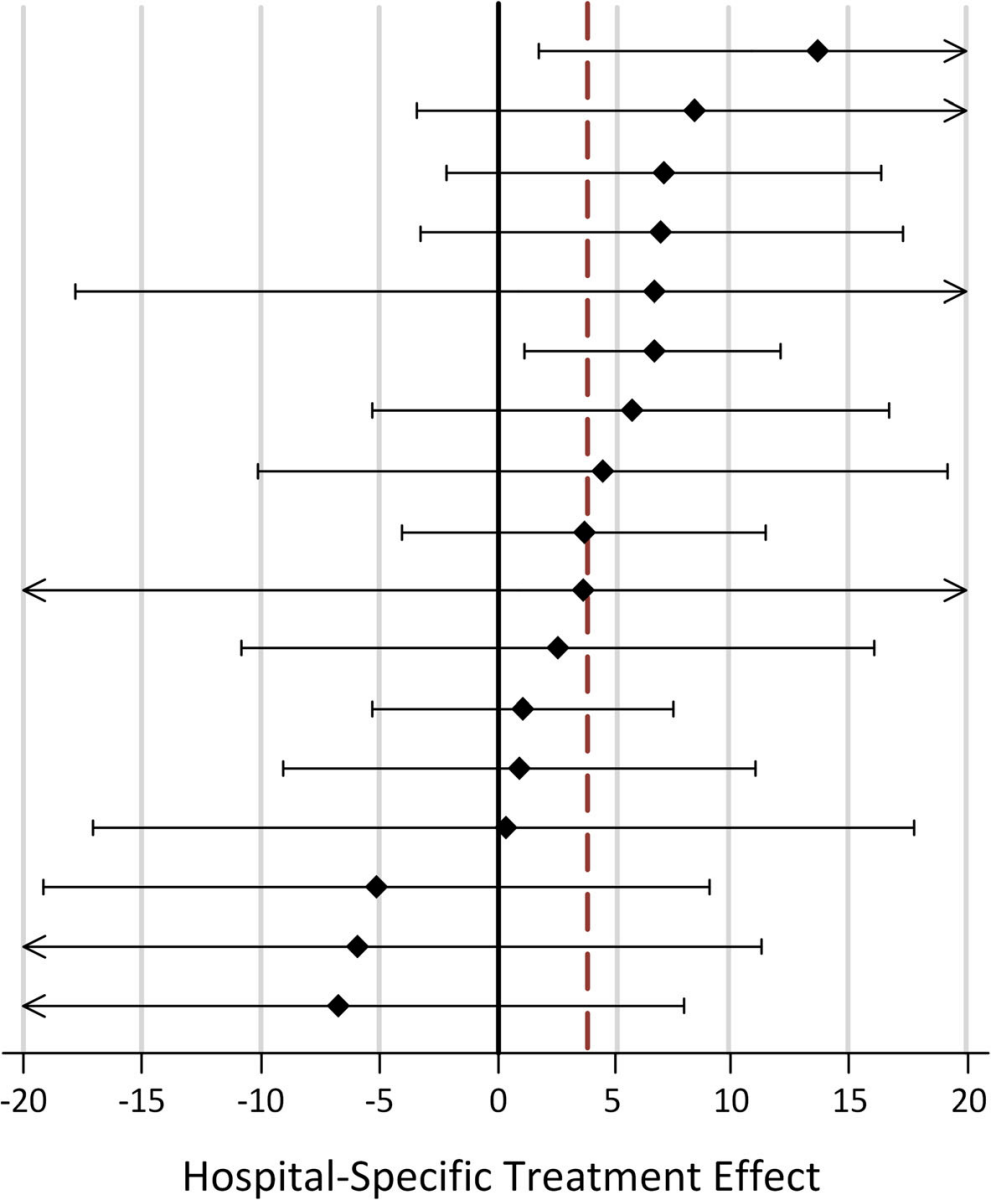
Within-Hospital Differences in Stroke Impact Scale (SIS)-16 between Patients Receiving a 14-day Visit. Forest plot of hospital-specific estimates and 95% confidence intervals (CI). Linear mixed models included propensity scores to account for differences in patients receiving the intervention versus not. Dotted line indicates the overall estimate in treated versus non-treated patients. CI values beyond ±20 are indicated with arrows

Mean SIS-16 among patients attending a visit within 14 days was 83.0 (SD 19.1) compared with 78.7 (SD 22.1) among those with no visit (adjusted mean difference 3.84 (95% CI 1.42 to 6.27, *p* = 0.002)). Hospital-specific estimates are shown in Fig. 5. The estimated difference in SIS-16 for patients who attended a clinic visit after 14 days (15–30 days) compared with non-attendees (5.35; 95% CI 0.76 to 9.94) was similar; however, we had limited sample size to evaluate visit timing.

### Maintenance

Fourteen of 19 (74%) hospitals sustained COMPASS-TC beyond Phase 1 for at least 6 months. Sustaining hospitals had higher partnership synergy than non-sustaining hospitals (median 4.1 vs. 3.0, *p* = 0.0067). No other measured hospital characteristics (Primary Stroke Center status, annual stroke volume, geographic region, academic affiliation, adequate backup for PAC and APP, PAC turnover, APP turnover, clinic setting, organizational readiness) significantly differed by maintenance status.

## Discussion

In the cluster-randomized pragmatic trial of COMPASS-TC, our analysis suggests that implementation of this comprehensive, evidence-based model of post-acute care that met TCM billing requirements was: (1) challenging; (2) multi-dimensional; and (3) impacted by both patient- and system-level factors. There was significant heterogeneity in hospital infrastructure, staffing, site performance, and diversity of the settings in which intervention was delivered. Delivering the care model in hospitals in rural areas and with higher organizational readiness was associated with successful implementation. Within-hospital analysis revealed a clinically meaningful difference in physical function when COMPASS-TC was received according to CMS standards of TCM billing compared with non-receipt of COMPASS-TC. The treatment effect did not diminish when visits occurred beyond the 14-day TCM window.

We employed several unique strategies to enhance COMPASS-TC use. First, it was designed, implemented, and continuously refined in collaboration with a broad range of stakeholder groups (stroke survivors, family caregivers, clinicians, advocacy organizations, community-based services, hospitals and health systems, industry partners, payers, and policy makers), which encouraged patient-centeredness and system-level buy-in for adoption [35]. Second, unlike many standard research studies, hospitals in this pragmatic trial used their own infrastructure, budget and staffing to deliver the intervention with a small per-participant stipend from the coordinating center. Third, we monitored quality metrics and provided monthly reports on these metrics to aid hospitals in their implementation-related continuous quality improvement (QI) efforts. Performance indicators are a commonly-used method of implementation of stroke quality of care [36, 37]. By using performance indicators, as we were evaluating the impact of the care model on patient outcomes, we were simultaneously evaluating implementation. These measures are the first to be established for post-stroke community-based care and could be an important foundation for future post-acute care QI efforts.

The RE-AIM framework is a widely-used and validated approach for assessing implementation [12]. Using RE-AIM, we found that, while hospitals overall had success with Adoption and Maintenance in COMPASS, Implementation and Reach were low. System-level characteristics associated with successful delivery of COMPASS-TC included meeting the patient in the hospital and scheduling the clinic visit before discharge. Another critical system-level characteristic was staff turnover rate, which impeded consistent delivery of the intervention. Organizational readiness, a modifiable system-level characteristic [38, 39], was positively associated with both Implementation and Reach. These findings suggest that effective Implementation will most likely occur at hospitals that already score high on readiness for organizational change or that create readiness before implementation. We found that increasing partnership synergy was associated with better Reach. Neurology-based clinics had greater Reach than other types of clinics, possibly because patients were more inclined to seek a stroke-trained specialist after discharge. Future research would need to investigate the effect of clinic type on patient decision-making and if these findings generalize outside of this study.

Patient factors influenced whether patients were reached by the intervention. Patient preference to seek follow-up care with providers they already had a relationship with, often primary care, was the most prevalent reason for clinic visit non-attendance. Stroke (versus TIA) diagnosis and having insurance were positively associated with clinic visit attendance, and history of stroke or TIA was negatively associated with attendance. Because the majority of patients were seen in specialty follow-up clinics, future implementation studies should identify strategies to address staffing and resource issues and to engage patients for specialty follow-up. The majority of patients in this study were seen in specialty rather than primary care clinics for follow-up. Studies are needed to identify the adequate financial and staffing resources required to fully engage stroke patients for follow-up and the ability of TCM reimbursement to support this level of care.

Other cluster-randomized trials of stroke interventions have been conducted, exhibiting different degrees of success with implementation and effectiveness. A trial of a stroke caregiver training strategy at discharge from the stroke unit in the United Kingdom (UK) showed no improvement in the primary outcome in the intention-to-treat analysis, similar to COMPASS, and the investigators noted variable uptake of the training strategy by the participating hospitals, with an average compliance of 44% [40]. A complex, highly effective intervention, ESD has been incorporated into standard care practices in the UK and Canada and has effectively reduced mortality and dependence among patients after mild to moderate stroke [41], but has not been adapted for the US healthcare system. In a qualitative assessment of implementation of ESD, there was clear buy-in and perceived benefits reported by inpatient and outpatient providers in both an urban and a semi-rural practice setting; however, challenges remained with integrating and streamlining care, specifically social care referrals [42]. An implementation analysis of a complex stroke rehabilitation trial, AVERT (A Very Early Rehabilitation for stroke Trial), also reported that successful implementation was due to interdisciplinary teamwork, education and stroke leadership to achieve buy-in, and developing different ways of working [43].

Our study has limitations. Small sample sizes limited our ability to precisely estimate associations between hospital and patient characteristics with Reach and Implementation of individual components of the intervention. Estimates of Effectiveness within intervention hospitals could be subject to unmeasured confounding, but propensity scores accounted for differences in important patient characteristics (e.g., stroke severity) between those treated and not treated within the same hospital. We are uncertain whether TCM was actually billed during the clinic visits; rather, we extrapolated this based on meeting the TCM billing criteria. Future analyses of administrative claims will inform how often TCM billing actually occurred. Implementation costs were not measured concurrently during implementation due to PCORI funding stipulations, but future analyses will evaluate implementation costs retrospectively through an ancillary funding source.

## Conclusion

Understanding the barriers and facilitators of COMPASS-TC implementation is essential for scaling and disseminating evidence-based, comprehensive TC to additional clinical settings. COMPASS-TC was designed to be consistent with CMS reimbursement requirements and the current US healthcare system. In this large-scale pragmatic trial, participating hospitals made substantial changes to both processes and structures of care to deliver COMPASS-TC within their everyday clinical practice using locally-determined hospital infrastructure and staffing. This systematic analysis of COMPASS-TC implementation informs how, and under which enabling contextual circumstances, this model of care may be effective. A future paper will describe the barriers and facilitators of implementation from the perspective of the front line providers.

### Supplementary information

**Additional file 1: Figure S1.** Days to Clinic Visit After Discharge. Histogram of percent of patients seen for follow up clinic by days after discharge.

**Additional file 2: Table S1.** Implementation Strategies Used in the COMPASS Cluster-Randomized Pragmatic Trial. Strategies used for the implementation of COMPASS.

## Data Availability

The datasets generated and/or analyzed during the current study are not publicly available due to the trial is ongoing and data sharing will follow the PCORI Policy for Data Access and Sharing but are available from the corresponding author on reasonable request.

## Quality Measure Definitions

### Telephone follow-up by day two

***Description*** *: Follow-up telephone call within two business days of hospital discharge or two attempts made*

**Denominator:**

Inclusion criteria:

- COMPASS eligibility criteria

Exclusion criteria:

- Patient hospitalized
- Patient transferred to a skilled nursing facility
- Patient deceased

**Numerator: The metric was met if any of the following occurred**

- Follow-up call completed within two business days
- Two attempts were made to complete the call
- Call was attempted, but not completed because the patient refused, could not complete, or did not have a workable number

### COMPASS clinic visit offered or scheduled by 14 days

***Description*** *: Receipt of follow-up visit within 14 days of hospital discharge or documentation that visit was offered/scheduled*

**Denominator**:

Inclusion criteria:

- COMPASS eligibility criteria

Exclusion criteria:

- Patient transferred to nursing home
- Patient hospitalized
- Patient deceased

**Numerator**: **The metric was met if any of the following occurred**

- Follow-up clinic visit completed by day 14
- Documentation of visit scheduled or offered but patient refused or did not show at the clinic

### Receipt of eCare Plan at a COMPASS TC visit

***Description*** *: Receipt of eCare plan during follow-up clinic visit*

**Denominator**:

Inclusion criteria:

- COMPASS eligibility criteria
- Attended a post-discharge clinic visit at any time after discharge

**Numerator**:

- eCare Plan generated electronically using the iPad or on paper and shared with the patient.

## Abbreviations

APP: Advanced practice provider
AVERT: A Very Early Rehabilitation for stroke Trial
CMS: Centers for Medicare and Medicaid Services
COMPASS: COMprehensive Post-Acute Stroke Services
COMPASS-TC: Comprehensive Post-Acute Stroke Services - Transitional Care model
CRN: Community resource networks
eCare Plan: Electronic care plan
ESD: Early supported discharge
GLMMs: Generalized linear mixed models
IRB: Institutional review board
NC: North Carolina
NP: Nurse practitioner
ORIC: Organizational Readiness to Implement Change
PA: Physician assistant
PAC: Post-Acute care coordinator
PCORI: Patient-Centered Outcomes Research Institute
PCP: Primary care provider
QI: Quality improvement
RN: Registered nurse
RUCA: Rural-Urban Community Area
SIS-16: Stroke Impact Scale-16
StaRI: Standards for Reporting Implementation Studies
TC: Transitional care
TCM: Transitional care management
UK: United Kingdom
US: United States
