# Supplementary figures and images for: Implementation of a billable transitional care model for stroke patients: the COMPASS study
Source: BMC Health Serv Res. 2019 Dec 19;19:978. doi: 10.1186/s12913-019-4771-0 (PMC6923985; doi:10.1186/s12913-019-4771-0)

**Supplemental Figure 1. Days to Clinic Visit After Discharge**


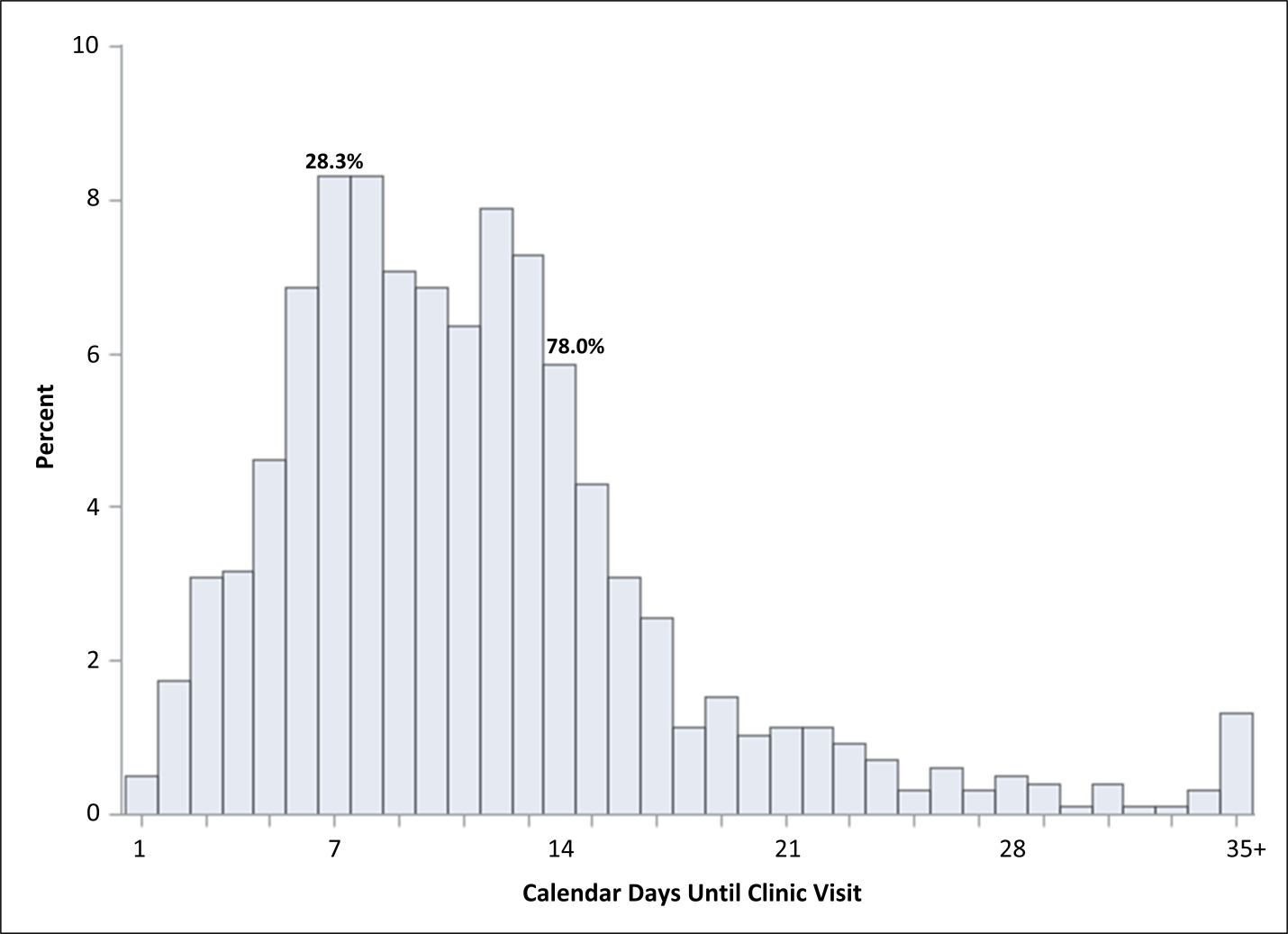

Supplement: Supplementary file 1 — Additional file 1: Figure S1. Days to Clinic Visit After Discharge. Histogram of percent of patients seen for follow up clinic by days after discharge. [file 12913_2019_4771_MOESM1_ESM.docx]
