# Supplementary material for: Implementation of a billable transitional care model for stroke patients: the COMPASS study
Source: BMC Health Serv Res. 2019 Dec 19;19:978. doi: 10.1186/s12913-019-4771-0 (PMC6923985; doi:10.1186/s12913-019-4771-0)
Supplement: Supplementary file 2 — Additional file 2: Table S1. Implementation Strategies Used in the COMPASS Cluster-Randomized Pragmatic Trial. Strategies used for the implementation of COMPASS. [file 12913_2019_4771_MOESM2_ESM.docx]

**Supplemental Table 1. Implementation Strategies Used in the COMPASS Cluster-Randomized Pragmatic Trial**

| **Strategy^18^** | **Specifications (which activities, by whom, when, how often)** |
| --- | --- |
| ***Prior to Enrollment of First Patient*** | |
| Assess for readiness and identify barriers and facilitators | - Assessed hospital factors that may impede or facilitate implementation of COMPASS-TC. - Director of Implementation had conference calls and/or hospital visits to identify strengths and available resources and to strategize on building a successful implementation team. - Described and assessed baseline standard of care via comprehensive survey. |
| Build a coalition | - Recruited and cultivated partnerships among hospitals, community providers, community-based organizations, and other key stakeholders, both locally, regionally, and statewide. - Implementation Team worked with hospitals to identify local healthcare providers and community resources (Area Agency on Aging for caregiver support programs, specialty pharmacies for medication management, evidence-based lifestyle interventions, FaithHealth, etc.) to build Community Resource Network to facilitate and support referrals. |
| Create new clinical teams | - Based on hospitals’ needs and resources, Director of Implementation helped hospitals build their clinical implementation teams, including the PAC, APP, and others with skills required to facilitate successful implementation. |
| Develop and implement tools for quality monitoring | - Building on the infrastructure of the North Carolina Stroke Care Collaborative stroke registry, created quality monitoring systems to identify eligible patients, capture baseline information, and to monitor intervention activities (2-day call, 14-day visit, 30- and 60-day calls) and to provide hospitals with monthly performance measure reports. |
| Develop educational materials | - Director of Implementation used a multidisciplinary team approach to create education and training materials and resources to facilitate clinicians’ understanding of and ability to deliver the COMPASS-TC model. Materials were user-friendly, written in comprehensible language, vetted by end-users, and discipline-specific (i.e., for PACs, APPs, Home Health and Outpatient Rehabilitation (HHOP) Providers, Physicians and Pharmacists, and Patients and Caregivers). - Presentations were created to accompany written materials, were professionally recorded in collaboration with the Area Health Education Centers (AHEC) system and were posted on the study website as enduring education and training resources. |
| Conduct educational meetings | - Meetings, conference calls, and webinars were held to teach stakeholders about COMPASS-TC, held for aggregate waves as hospitals were launched. - Before launch, each hospital’s clinical team (PAC, APP) attended a two-day “boot camp” intensive training on COMPASS-TC where the Implementation Team shared discipline-specific presentations on various aspects of the model and facilitated hands on breakout sessions for practicing lessons learned. - Following boot camp training, each hospital received a site-specific day-long meeting facilitated by the Director of Implementation and members of her team to continue to prepare the hospital team and its Community Resource Network for launch. |
| Identify and prepare champions | - Identified champions through hospital system and by working collaboratively with statewide home health agencies and outpatient rehabilitation facilities. - A team of experts by discipline created written, voice-over-PowerPoint and video educational presentations on COMPASS-TC and Movement Matters Activity Program (MMAP) for Stroke, one-page patient and provider “Matters” documents on physical activity guidelines, cognition and insurance developed by the HHOP Engagement and Implementation Committee leaders. |
| Use train-the-trainer strategies | - HHOP champions were educated on the MMAP and instructed to use a train-the-trainer model to educate their front-line providers. |
| ***During Implementation of COMPASS-TC*** | |
| Audit and provide feedback | - Data Team, with assistance from Implementation Team, conducted two unannounced case ascertainment audits, each covering a two-month period, to ensure all eligible patients were enrolled. Implementation Team shared information with hospitals on areas of missingness and improvement needed. |
| Conduct educational meetings | - Hospitals participated in monthly site-specific educational meeting with Director of Implementation for ongoing education and training in identified areas of need. |
| Facilitate relay of clinical data to providers | - In the educational meetings (described above), Director of Implementation shared monthly review of the site’s performance measure report to track progress on improvement, compare with peer hospitals’ performance, strategize about how to continue to improve, and give positive feedback on successes. |
| Conduct educational outreach visits | - Additional site-specific visits made upon request or as identified as necessary by Director of Implementation (e.g., during staff turnover) to train on COMPASS-TC in the practice setting. |
| Create a learning collaborative | - Created two statewide learning collaboratives, one for the clinicians implementing COMPASS-TC and one for HHOP therapists to whom COMPASS patients were referred:  1. Created a coalition of PACs and APPs and fostered co-learning and joint problem solving. Once a hospital started implementing COMPASS-TC, monthly site-specific calls with the Director of Implementation began as well as bimonthly conference calls and webinars with their wave aggregate to continue their education and training. Each wave received a survey in advance of their aggregate meeting, and the results were used to drive the agenda for these meetings. Topics discussed included administrative challenges, challenges with identifying eligible patients, challenges with the 2-day follow up call, challenges with the 14-day clinic visit, etc. Webinars were led by the Director of Implementation. After 3 months, members of the wave aggregate were surveyed to determine if they felt ready to transition to monthly educational training webinars. 2. Created a coalition of HHOP Rehabilitation therapists and fostered co-learning and joint problem solving. Bimonthly and transitioning to monthly teleconferences, facilitated by HHOP Engagement and Implementation Committee leaders; webinars by field experts on pertinent topics in stroke (e.g., Healthy Aging North Carolina, Cognitive Aspects of Stroke); surveys administered prior to each call and at 90 days and 1 year on adoption and implementation of the Movement Matters Activity Program for Stroke. |
| Identify early adopters | - Identified early adopters to learn from their experiences. - Director of Implementation and her team monitored hospitals, their HHOP teams, and local community-based agencies for evidence of communication, collaboration, and participation in learning collaboratives, and by progress on their performance measure reports. - Early adopters assisted the Implementation Team with educating and training other hospitals at subsequent boot camps and during bimonthly to monthly education and training webinars. |
| Provide local technical assistance | - Technical assistance was often needed for issues with the data application collection tool and eCare Plan generation. Members of the Implementation Team monitored their e-mail daily and provided numbers for clinical team members to call when urgent technical assistance was needed. |
| Provide ongoing consultation | - The Implementation Team was available by e-mail and office and personal phones for immediate questions or ongoing consultation to support implementation of COMPASS-TC. |
| Tailor strategies | - During site-specific conference calls, and by reviewing monthly performance measure reports, the Director of Implementation and her team worked with hospitals on an ongoing basis to tailor their processes for implementation until a successful system was in place. |
